# Thank you to our 2025 reviewers

**DOI:** 10.1016/j.lana.2026.101421

**Published:** 2026-02-13

Rahi Abouk

Dmitry Abramov

Gabriella Abreu

Osama Ali Abulseoud

Tim Adair

Victor Adekanmbi

Miguel E Aguado-Barrera

Allison Aiello

Joel Aik

Mona S al-Ahmad

Ana Alastruey-Izquierdo

Harold Alderman

Margarita Alegria

Gregory L Alexander

Victoria Allgar

Othman Al-Sawaf

Flávia Jôse Oliveira Alves

Hany Aly

Juan Carlos Alzate-Ángel

Robert Ancuceanu

Benjamin Anderson

David M Anderson

Monica Andrade

Melissa Andrew

Martin Angel

Francisco E Arancibia

Catalina Arango-Ferreira

Lucio Araujo

Pedro Arcos González

Carmen Ardanuy

Nidia Aréchiga-Ceballos

Gloria Arriagada

Mattia Arrigo

Catherine Arsenault

Rasha Ashmawy

Sachin Atre

Brandon K Attell

Beatriz Paulina Ayala Quintanilla

Juan Jose Badimon

Sandra Baez

Rodrigo Bagur

Janak Bahirwani

Manisha Bahl

Judit Bajor

Anita Balakrishnan

David Baldwin

Ovidiu Constantin Baltatu

Siddhartha Bandyopadhyay

Eugenio Baraldi

Lorena Barberia

Whitney Barnett

Jenny Barrett

John Bartkowski

Alfred Bartolucci

Joao Bastos

Julian Baudinet

Sherry Beaudreau

Martin Beaulieu

Tatiana Benaglia Carvalho

Pedro Benetti

Corina Benjet

Rebecca Bentley

Zombor Berezvai

Carol Berkowitz

Antonio Bernabe-Ortiz

Filipe Andrade Bernardi

Wanderley Marques Bernardo

Astrid Bertsche

Julie Bettinger

Hind A Beydoun

Praveen Bharti

Sonu Bhaskar

Nazim Bhimani

Shilpa N Bhupathiraju

Burcu Kucuk Bicer

Merijn Bijlsma

Sheila Bird

Courtney Blackwell

Marco H Blanker

Cari Bogulski

Raquel Regina Bonelli

Amelie Bonnefond

Victor Borja-Aburto

Anne E Boustead

María Belén Bouzas

Erick Boy

Adam Bramoweth

Ola Bratt

Paul Brennan

Luís Fernando De Macedo Brígido

Fraser Brims

Matthijs Brouwer

Adrian Brown

Adam Brufsky

Aline Bruni

Pierre Buekens

Romina Buffarini

Caroline Busatto

Julie Buser

Patricia Bustos

Siddappa Byrareddy

Alfonso Cabello

Carlos Caceres

Ahmet Okay Caglayan

Monica Caicedo-Roa

Gustavo Yano Callado

Gabriela Callo-Quinte

Jackie Campbell

Janis Campbell

Jeffrey I Campbell

Mabel Carabali

Claire Carette

Robert G Carlson

Andre Carmo

Ben Carter

Paulo Carvalho

Marcia Castro

Bess L Caswell

Igor Cavallini Johansen

Marina G Cavuoto

Jude Mary Cénat

Thiago Cerqueira-Silva

Aïna Chalabaev

Katrina Champion

Huan Keat Chan

Julia Yu Fong Chang

Kath Checkland

Hiam Chemaitelly

Diego Chemello

Haiquan Chen

Ingrid Chen

Mu-Hong Chen

Ka Shing Cheung

Silvia Chiang

Sonja Chiappetta

Dipanjan Chowdhury

José Miguel Cisneros

Philip Clare

Kristopher Clark

Victoria Clarke

Tammy Clifford

Kalysha Closson

Alyssa Cohen

Anna Conway

Andrew Copas

Vladmir Cláudio Cordeiro de Lima

Rosie Cornish

Juan Corral

Adolfo Correa

Fiammetta Cosci

Linda Cottler

Paul Cottu

Douglas E Crews

Julio Croda

Maria Sofia Cuba-Fuentes

Anthony Culyer

Amanda Cunha

Mariana Cunha

Paula Cunnea

Steve Cunningham

Maria Curado

Wayne Cutfield

Salvatore Lucio Cutuli

Thuane Huyer Da Roza

Jesse Lopes da Silva

Timothy P Daly

Rodolfo Damiano

Carlton D Dampier

Mary Danforth

M Carolina Danovaro-Holliday

Pojsakorn Danpanichkul

Mandira Daripa Kawakami

Nicholas Davies

Héber Salvador C de Castro Ribeiro

Patrícia F de Fragas Hinnig

Inge de Kok

Fernando de la Hoz-Restrepo

Lucas Vinícius de Lima

Priscila de Morais Sato

Giuseppe De Palma

William Marciel de Souza

Shihan Deng

Qian Di

Patricia Di Ciano

Antonio Di Sabatino

Daniel Diaz

Cristian Diaz-Velez

Joseph Dieleman

Kirsten Dillon

Dobromir Dimitrov

Kiril M Dimitrov

Tao Ding

David Dingli

Donald DiPette

Kevin K Dobbin

Matthew Dodd

Christyn L Dolbier

Jianzeng Dong

Achsah F Dorsey

Kylie Dougherty

Jan Felix Drexler

Lingbin Du

Julien Dumurgier

Lisa M Dunkle

Andre Duraes

Doris Duran

Christine Durand

Laura Dwyer-Lindgren

David T Dyjack

Kirk Easley

Ikenna Ebuenyi

Sandrah Eckel

Sarah Edwards

Andrey Egorov

Gideon Emukule

Shymaa Enany

Cornelia Englisch

Benjamin Enns

María Fernanda Escobar-Vidarte

Babak Eshrati

Manuel Antonio S Espinoza

Beverley Essue

Maria Estevez-Diz

Fernando Estévez-López

Paolo Eusebi

Stephanie Evans

Lee Evelyn

Valeria Fabre

Oluwaseun Falade-Nwulia

Irene Falgas-Bague

Yun Fan

Chi-Tai Fang

Eric Faragher

Forough Farrokhyar

David Fei-Zhang

Michelle Fernandez

Carlos Gil Ferreira

Donna Ferriero

Erika Fialho

Francesca Mapua Filbey

Kate Filia

Tommaso Filippini

Katharina Fink

Lucy Finnigan

Carlo Fischer

Emily R Fisher

Erin Fleischer

Vagner Fonseca

Nathan P Ford

Nicole Ford

Andre Ricardo Ribas Freitas

Anna Freni Sterrantino

Francesca Frentiu

Joseph Friedman

Madeline Frost

Hongqiao Fu

Bruno Fuchs

Ary Gadelha

Carlos Grabois Gadelha

Kenny Mauricio Gálvez

Leila García

Claudia Garcia Serpa Osorio-de-Castro

Miguel Angel Garcia-Bereguiain

Graham Gardner

Richard Garfein

Katherine A Garnham

María Garzón

Rebecca Geary

Tebeb Gebretsadik

Alison Gemmill

Sommer E Gentry

Marco Geraci

Kathryn Gex

Daniele Roberto Giacobbe

Andrea Giacomelli

Luca Giannella

Theodoros V Giannouchos

Shannon L Gillespie

Richard Gillum

Stuart Gilmour

Antonio Gil-Ugidos

Emanuele Giorgi

Marta Giovanetti

Federico Gobbi

Dawn A Goddard-Eckrich

Peter Goldblatt

Shira Goldenberg

Delia Goletti

Fabio Gomes

Anthony Gonçalves

Marcelo Gonçalves

Alessandro Gonfiotti

Jose L Gonzales

Alfonso A Gonzalez-Valderrama

David Goodman-Meza

Steven Gortmaker

Carolyn Gould

Brigid K Grabert

Amanda Graham

David Graham

Brittany Greene

John Gregson

Lionel Gresh

Anthony Griffin

María Eugenia Grillet

Martin Grobusch

Amos Grunebaum

Eduard Guasch

Nathalia Guimarães

Raphael Guimarães

Ruth Guinsburg

Bashar Gulumbe

Samir Gupta

Adrian Guta

César Gutiérrez Villafuerte

Terry Haines

Wayne Hall

Catriona Halliday

Eugenia Haluszka

Luciana Hannibal

Bo Terning Hansen

Thomas Harder

Michael Harhay

Diane Harper

Ché Matthew Harris

Patrick Harris

Mahmoud M Hassanein

Jinbo He

Yulei He

Craig W Hedberg

Fernando Hellmann

Lisa Henriksen

Juan Hernandez-Avila

Juan Herrera-Escobar

Attila Hertelendy

Amelia Hessheimer

Patrice Hicks

Briony Hill

Yeh-Li Ho

Martin Hoenigl

Richard M Hoffman

Gladys N Honein-AbouHaidar

Namki Hong

Amy P Hsu

Li Hu

Qing-Hai Hu

Jee-Fu Huang

Yueqin Huang

Celia Hubert

Megan Huchko

Amanda Hughes

Nathalie Huguet

Sally Hunsberger

Luke Hunter

Emily Hurley

Gerard A Hutchinson

Clara Hwang

Usman Nurudeen A Ikumapayi

Ana-Maria Iosif

Eddie Irizarry

Stephanie Irving

Pedro Henrique Isaacsson-Velho

Maria Izquierdo-Pulido

Alessandro Jatobá

Bec Jenkinson

Majken Karoline Jensen

Seogsong Jeong

Katarzyna Jerzak

Fanpu Ji

John Ji

Clarisse Joachim

Candice Johnson

Dayna A Johnson

Emily E Johnston

Sarah Elizabeth Jolley

Rolf Jorde

Boyoung Joung

Lucia D Juarez

Steven Julious

Jihoon Jung

Monica Jung

Mikk Jürisson

Didier Jutras-Aswad

Anna Kågesten

Anna Kalbarczyk

Mona Kanaan

Anita Kar

Shama D Karanth

Johanna Kattenberg

Jay Kaufman

Marissa Kellogg

Nicole Kelly

Mark Kelson

Zoe Kelson

Ulrike Kemmerling

Stephen Kerr

Robert Stephen Kerrison

Roxanne Keynejad

Ali Khashan

Rachel Kidman

Dae Jung Kim

Dajung Kim

Daniel Kim

Isaac Yi Kim

Michelle Kang Kim

Raymond Kim

Carina King

Martin King

Ole Kirk

Theo N Kirkland

Masatoshi Koga

Nils Kok

Dunja Kokotovic

Qingzhou Kong

Evangelos Kontopantelis

Srinivas Kota

Charles Kouanfack

Christian Kratz

Bernardo Javier Krause

Noa Krawczyk

Alexander Kreuter

Partha Krishnamurthy

Daniel J Kruger

Leighton Ku

Michael Kucharczyk

Alexandros Laios

Sham Lal

Grace Lam

Gabriel Laporta

Justin R Lappen

Regina LaRocque

Lisa R LaRowe

Phyu Mon Latt

Eric Lavigne

Daniel Laydon

Onício Batista Leal-Neto

Monique Lechenne

Whanhee Lee

Yi-Chia Lee

Jiayao Lei

Stephanie A Leonard

Ganna M Leonenko

Arash Letafati

Maddalena Lettino

Wai Leung

John Leventhal

Jie Li

Jingxin Li

Tim M H Li

Wenhui Li

Yongze Li

Ziyu Li

Shih-Cheng Liao

Cuauhtemoc Licona Cassani

Jue Tao Lim

Sheila Lima

Chihchuan Lin

Tracy Lin

Karolina Linden

Anthea Lindquist

Buyun Liu

Jifeng Liu

Jue Liu

Ronggao Liu

Xiaoxue Liu

Yiran Liu

Andrea Llera

Jamie Oi Ting Lo

Christine T Loftus

T K Logan

Fabian Lohaus

Anna Lok

Norma Lopez Facundo

Julieta López Vázquez

Andrés López-Cortés

José Francisco López-Gil

Patricio Lopez-Jaramillo

Xavier Lopez-Labrador

Geidy Lorenzo

Alessandra Lugaresi

Hao Luo

Audrey Lyndon

Suzanne MacFarland

Ísis Eloah Machado

Eleni Magira

Gabriela Tavares Magnabosco

Alyson L Mahar

Mathieu Maheu-Giroux

Henry Maia Peixoto

Edward Maibach

Marcia Makdisse

Jean Makuza

Narmeen Mallah

Corinne Mandin

Mattia Manica

Lilli Mann-Jackson

Mohammad Ali Mansournia

Limin Mao

Frances A Maratos

Paola Marchesini

Silvana Marchioro

Têmis Maria Félix

Tina M Marinelli

Michael Marks

Alexandre Rodrigues Marra

Mauro Marrelli

Zack Marshall

Alexandro Martagon

Molly Martin

Marianne Martinello

Ramon Martinez

Pablo Martinez-Camblor

Poliana Cardoso Martins

Francisco Rogerlândio Martins-Melo

Maryann Mason

Guilherme Mataveli

Elaine Fernandes Mateus

Fernanda Matozinhos

Abigail Matthews

Delvon T Mattingly

Safouane Maurens-Hamdi

Izabela Maurício de Rezende

Walter Mazzucco

Jennifer M McAllister

Stephen McCall

Dennis McCarty

April N McDougal

Tara A McKay

Robert McLellan

Brendan Joseph McMullan

James H Mehaffey

Madeline Meier

Christian Mejia

Andreia Melo

Cristian Mena

Jeremy Mennis

Fiona Mensah

Anwar Merchant

Stephanie Merhar

Claudio Tinoco Mesquita

Sarah Messiah

Jane M Metrik

Rachel Miller

Paula Lana de Miranda Drummond

Guadalupe Miranda-Novales

Biswadev Mitra

Seyed Moghadas

Bruce D Moore

Harriet Elizabeth Moore

Gláucia Maria Moraes Oliveira

Carla Moreira

Rogeria Moreira

Carolina Moreno

Rosemary Morgan

Elaine Morrato

Matthew P Motta

Seyed Ehsan Mousavi

Pricila Mullachery

David Muscatello

Vincent Mysliwiec

Lior Naamati-Schneider

Mathieu Nacher

Mahdi Naeim

Atsuko Nakatsuka

Massimo Nardone

Gustavo G Nascimento

Sarah Nash

Ahmed Nasreldein

Daniel E Neafsey

Francesco Negro

Josef Neu

Mytien Nguyen

Federico G Nicola

Susan Niermeyer

Nikita Nikita

Dariya Nikitin

Georges Noël

Ana Maria Nogales Vasconcelos

Leticia Nogueira

John Norrie

Bernard North

Seyed Mehdi Nouraie

Amy Nunn

J D Ny

Hiroshi Ohtsu

Kazunori Oishi

Torsten Olbers

Antonio Olivas-Martinez

Laura Oliveira

Jamary Oliveira-Filho

Hanna Oltean

Colm O'Morain

Camila E Orsso

Jovita Ortiz-Contreras

Esteban Ortiz-Prado

Kevin Outterson

Carmencita David Padilla

Gabrielle Page

James Paik

Enny Paixao

Romain Palich

Amanda C Palmer

Jay Pan

Dimitrios P Panagoulias

Basu Pandey

Elena Pariani

Jean-Jacques Parienti

Jose Parodi

Luke Parry

Patrizio Pasqualetti

Pragna Patel

Nikhilesh G Patil

Marcos Pascoal Pattussi

Alex Pauvolid-Corrêa

Janine Paynter

Lillian Pazvakawambwa

Marcella Pazzinatto

Felicidade Pereira

David Perez

Ana Pérez

Gonzalo Perez Marc

Eliseo Pérez-Stable

Henry Perry

Ponni V Perumalswami

Julia Pescarini

Raphael Manhaes Pessanha

Vitor Pessoa Colombo

Olumuyiwa James Peter

Michael Peters

Pammla Petrucka

Ciaran S Phibbs

Giorgina Barbara Piccoli

Mercedes Pilkington

Paulo Pinheiro

Yago Tavares Pinheiro

Mariantonietta Pisaturo

Marissa H Platner

Robert Podolsky

Harold Pollack

Marija M Polovina

Maximiliano Loiola Ponte de Souza

Allison Portnoy

Cristina Poveda

Fynnwin Prager

Robin Prescott

Klaus Prettner

Miguel Prieto

German Prieto-Rodriguez

Elizabeth Prince

Jan Prins

Simon Procter

Luise Pruessner

Maria Pyra

Ibrahim Qaddoumi

Jin Wei Qiang

Guoyou Qin

Boris Quednow

Henry C Quevedo

Carlos Quispe-Vicuña

Kenneth Rabin

Benjamin M Rader

Claudia Rafful

Thaynã Ramos Flores

Julia Raney

Otavio Ranzani

Prateek Rastogi

Maurianne Reade

Dinah Reddihough

William Reisen

Christel Renoux

Weeberb Requia

Heloisa Resende

Aaron Reuben

Paul Revill

Luz Reynales-Shiguematsu

Michael E Rezaee

Suely Meireles Rezende

Antonio Ribeiro

Rodrigo Ribeiro-Rodrigues

Natalie Riblet

Henry Rice

Marcus Richards

Ralph Riello III

Alan S Rigby

Juvenal A Ríos

Ligia Estefanía Rios López

Pamela Michelle Cornejo Rivas

Jairo Rivera

Jason D Robinson

Teresita Rocha-Jimenez

Hector Rodriguez

Isabel Rodriguez Barraquer

Danae Rodríguez Gatta

Alfonso J Rodriguez-Morales

Ashley Roen

Leonardo Roever

Julia Rohr

Camila Romano

Zheng Rongshou

Regis Rosa

Jonathan Rosand

Adriana Beatriz Rose

Gholamreza Roshandel

Perran A Ross

Alexandre Tellechea Rotta

Soumyajit Roy

Yibing Ruan

Christiana M Russ

Graciela Russomando

Elizabeth Ryan

Claude Sabeta

Martha Idalí Saboyá

Marc Saez

Taha Koray Sahin

Eiko Saito

Arghavan Salles

Vafi Salmasi

Laura Samuel

Javier Sánchez

Ronald Sanders Jr

Thomas Santo

Alan Roger Dos Santos-Silva

Alexandra Savinkina

Ambreen Sayani

Fernanda Scagliusi

Alejandro Schijman

Julia P Schleimer

Gabriel Schnitman

Tim Schubert

Lavinia Schuler-Faccini

Christina Schumacher

Lisa M Schumaker

Naomi Schwartz

Sylvain P Sébert

Akihiro Seita

Daniel Semenza

Carlo Senore

Edward Septimus

Victor Sequera

Katie Severn

Rowland Seymour

Robert Shafer

Morteza Shamsizadeh

Nadim Sharif

Amin Sharifan

Monisha Sharma

Fadia Shaya

Muki Shey

Ju-Fang Shi

Kwang Ho Shin

K M Shivakumar

Arjumand Siddiqi

Monica Sierra

Juan Carlos Silva

Hannah A Silverstein

Jason Silvestre

Stephanie Sinclair

Laramie Smith

Rebeccah Sokol

Dmitriy Sonkin

Susan C Sonne

Sveinung Sørbye

Antoni Soriano-Arandes

Álvaro Francisco Lopes De Sousa

Dyego L B Souza

Kerstin Spanhel

Igor Spiroski

Tanja Stamm

Bridget Steele

David A Stempel

Philipp Sterzer

Garrick C Stewart

Jason Stout

Ramnath Subbaraman

Yi Nam Suen

Mina Suh

Jing Sun

Yingxian Sun

Kena A Swanson

Din Syafruddin

Hsing Hua Sylvia Lin

Stanley Szefler

Howard Takiff

Andrew H Talal

Byomkesh Talukder

Xiao Tan

Kenichi Tanaka

Weiming Tang

Christopher Taylor

Mark Taylor

Roy Taylor

Chloe Teasdale

Yacob G Tedla

Helena Teede

Anastasios Tentolouris

Martha K Terris

Per M Thorsby

Luiz Claudio Thuler

Sílvia Maria De Oliveira Titan

Boghuma Titanji

Erling Tjora

Michael Tong

Julio Torales

Carlos Toro-Huamanchumo

Els Torreele

Laura Torres-Collado

Marcos Roberto Tovani-Palone

Gabriel Trueba

Christos Tsagkaris

Lilian Rumi Tsuruta

Wen-Jun Tu

Bernardo Tura

Rodman Turpin

Emre Umucu

Eduardo Undurraga

Juan Undurraga

Luissa Vahedi

Fernando Val

Yamile Valdes Gonzalez

Sergio Valdés-Ferrer

Diama Vale

Pablo Valente

Bert Jan H van den Born

Carol Ann van Hulle

Mark Varvares

Sonia Vasconcelos

Elizabeth Vaughan

Flavio Veras

Terril Verplaetse

Alejandro Videla

Carlos Cezar Flores Vidotti

Randy A Vince

Andrea Visentin

Erin Vogel

Alex Vorsters

Roberta L Waite

Wayne W Wakeland

Etienne Waleckx

Wendell Codrington Wallace

Alexander Walley

Heather M Walline

Per Wandell

Jianing Wang

Jianming Wang

Minmin Wang

Tiange Wang

Yi Wang

Yu-Ming Wang

Susitha Wanigaratne

Kathleen Wannemuehler

David Watkins

Mark Weatherall

Marcia Weaver

Rae Ellen Webb Kavey

Talia Wegman-Ostrosky

Wei Wei

Arved Weimann

Claudia Weiß

Jordan Weiss

Paul Weiss

Hilary Wething

Cassandra White

Sarah Whitton

Emerson Wickwire

Andrew M Williams

Ariel A Williamson

Jack Wilson

Nina Wilson

T Charles Witzel

Judith Ju Ming Wong

Jilei Wu

Qinan Wu

Sangang Wu

Yan-Ting Wu

Katarzyna E Wyka

Melissa Shepanski Xanthopoulos

Jingyi Xiao

Shangzhi Xiong

Beibei Xu

Chengdong Xu

Xiaonan Xue

Hemang Yadav

Flávia Yoshie Yamamoto

Alice Fang Yan

Juan Yang

Tingyu Yang

Yang Yang

Mary Yannakoulia

Pengpeng Ye

Xin Ye

Nirupama Yechoor

Eng-Kiong Yeoh

Jianhai Yin

Peng Yin

Vignan Yogendrakumar

Sang Gune Yoo

April Young

Hossein Zare

Carol Zavaleta-Cortijo

Rachel A Zeig-Owens

John M Zempel

Fengqing Zhang

Junfeng Zhang

Wangjian Zhang

Xiaoxi Zhang

Xueying Zhang

Ying Zhang

Fengmin Zhao

Shi Zhao

Miaobing Jazzmin Zheng

Bin Zhou

Rongrong Zhou

Xiao-Nong Zhou

Linnea A Zimmerman

Gianvincenzo Zuccotti

Zachary Zumsteg

